# Do early father–infant interactions predict the onset of externalising behaviours in young children? Findings from a longitudinal cohort study

**DOI:** 10.1111/j.1469-7610.2012.02583.x

**Published:** 2012-07-19

**Authors:** Paul G Ramchandani, Jill Domoney, Vaheshta Sethna, Lamprini Psychogiou, Haido Vlachos, Lynne Murray

**Affiliations:** 1 Department of Psychiatry, University of Oxford, Warneford HospitalOxford, OX3 7JX, UK; 2 Milton Keynes Community Health ServicesStanding Way, Eaglestone, Milton Keynes, MK6 5NG, UK; 3 Winnicott Research Unit, School of Psychology and Language Sciences, University of ReadingReading, RG6 6AL, UK

**Keywords:** Child behaviour, parent-child interaction, fathers

## Abstract

**Background:**

Factors related to parents and parenting capacities are important predictors of the development of behavioural problems in children. Recently, there has been an increasing research focus in this field on the earliest years of life, however, relatively few studies have addressed the role of fathers, despite this appearing to be particularly pertinent to child behavioural development. This study aimed to examine whether father–infant interactions at age 3 months independently predicted child behavioural problems at 1 year of age.

**Method:**

A sample of 192 families was recruited from two maternity units in the United Kingdom. Father–infant interactions were assessed in the family home and coded using the Global Rating Scales. Child behaviour problems were assessed by maternal report. Hierarchical and logistic regression analyses were used to examine associations between father–infant interaction and the development of behavioural problems.

**Results:**

Disengaged and remote interactions between fathers and their infants were found to predict externalising behavioural problems at the age of 1 year. The children of the most disengaged fathers had an increased risk of developing early externalising behavioural problems [disengaged (nonintrusive) interactions – adjusted Odds Ratio 5.33 (95% Confidence Interval; 1.39, 20.40): remote interactions adj. OR 3.32 (0.92, 12.05)]

**Conclusions:**

Disengaged interactions of fathers with their infants, as early as the third month of life, predict early behavioural problems in children. These interactions may be critical factors to address, from a very early age in the child’s life, and offer a potential opportunity for preventive intervention.

## Introduction

Behavioural disorders are the commonest psychological problem affecting children. They are associated with a wide range of poor outcomes in adolescence and adult life, including academic failure, delinquency, peer rejection and poor psychiatric and physical health (Campbell, 1995; Scott, Knapp, Henderson, & Maughan, 2001; Shaw & Gross, 2008). These outcomes represent a considerable health and social burden, with high levels of cost to society (Scott et al., 2001). It is becoming increasingly apparent that the roots of enduring behavioural problems often lie in early life, and the trajectories of behavioural problems often extend back into the preschool years (Shaw, Gilliom, Ingoldsby, & Nagin, 2003; Tremblay et al., 2004).

The essential symptoms of behavioural problems are identifiable from a young age (Petitclerc & Tremblay, 2009). One of the difficulties of determining the clinical significance of behaviour problems in young children is that many of the behaviours of interest (e.g. tantrums, noncompliance and aggression) are also normative behaviours during this period, and it is the extent and persistence of these problems that are the important distinguishing characteristics. Oppositional and aggressive behaviours, such as hitting and biting can start from as early as 12 months of age and increase significantly in the second year of life (Alink et al., 2006; Tremblay et al., 2004). These problems often peak at the end of the second year, and diminish afterwards, in part due to socialisation effects and the development of alternative ways of managing conflict within families. However, not all children follow this pattern. Several studies have found that a significant minority of children, around 6% of normative samples, show persistent oppositional and aggressive behaviour across childhood (e.g. (Moffitt & Scott, 2008; Nagin & Tremblay, 1999; Shaw et al., 2003). A review of community and primary care studies of children aged 2–5 years (Egger & Angold, 1996) concluded that the proportion of children meeting diagnostic criteria for behavioural problems was between 2% and 6% for ADHD, 4 and 8% for Oppositional Defiant Disorder and 0 and 4% for Conduct Disorder. Furthermore, children with the highest levels of oppositional behaviours in the preschool period show higher levels than their peers into adulthood, although it should be noted that most studies to date have shown continuities from the age of 2 or 3 years onwards rather than earlier (Caspi, Moffitt, Newman, & Silva, 1996; Moffitt & Scott, 2008).

Epidemiological studies have identified a number of risk factors for the onset and continuity of behavioural problems (e.g. (Moffitt & Scott, 2008; Petitclerc & Tremblay, 2009). Amongst these, parenting characteristics and patterns of parent-child interaction are of key importance as they are strongly and consistently identified as risk factors, with adverse risk associations continuing into adult life (Loeber, Burke, & Pardini, 2009). They are also amenable to clinical intervention [e.g. (Webster-Stratton, Reid, & Hammond, 2004)]. Studies of parental factors to date have often focussed on maternal factors (Tremblay et al., 2004). For example, maternal depression and negative mother-infant interactions (particularly decreased maternal sensitivity or responsiveness) predict children’s externalising problems in the preschool years (Miner & Clarke-Stewart, 2008; Owens & Shaw, 2003; Petitclerc & Tremblay, 2009). Parents’ skills at managing coercive interactions can influence the development of conduct problems, with positive parenting practices exerting effects that are independent of negative parenting (Gardner, Dishion, Shaw, & Burton, 2007).

## Father involvement

Although research in recent years has increasingly explored the role of fathers in children’s development (Lewis & Lamb, 2003), it has been relatively neglected in terms of early child development. Evidence suggests that mothers and fathers interact differently with their children from an early age, with fathers spending a higher proportion of their interacting time in play than mothers, and fathers’ style of interaction being more physically stimulating and unpredictable than mothers (Lewis & Lamb, 2003). Furthermore, a limited number of studies suggest that fathers may contribute uniquely to children’s early behavioural development, including the development of behavioural problems, over and above the effect of mothers (DeKlyen, Biernbaum, Speltz, and Greenberg, 1998; DeKlyen, Speltz, and Greenberg, 1998; Lewis & Lamb, 2006; Ramchandani, Stein, Evans, & O’Connor, 2005). Paternal psychopathology, including depression (Paulson, Dauber, & Leiferman, 2006) and antisocial personality traits (Jaffee, Moffitt, Caspi, & Taylor, 2003), is also associated with an increased risk of behavioural problems in offspring.

Paternal sensitivity in interactions with their young children predicts better behavioural and psychological outcomes for children later in development (Grossman et al., 2002; Trautman-Villalba, Gschwendt, Schmidt, & Laucht, 2006), whereas relationships that lack warmth or are rejecting can have a negative impact on behaviour (Amato & Rivera, 1999). Fathers’ involvement with their children’s lives has consistently been shown to influence child outcomes. Flouri and Buchanan (Flouri & Buchanan, 2004) found that father involvement predicted improved cognitive outcomes later in life, whereas Amato and Rivera (Amato & Rivera, 1999) showed positive father involvement to be associated with lower levels of child behaviour problems, even when mothers’ involvement was controlled for. A systematic review (Sarkadi, Kristiansson, Oberklaid, & Bremberg, 2008) suggested that fathers’ engagement with their child, as defined by direct contact such as play or care-giving, predicted positive outcomes. These included reduced behavioural problems in boys and emotional difficulties in girls, and reduced levels of delinquency in families from low resource backgrounds. Finally, DeKlyen and colleagues (DeKlyen, Biernbaum, et al., 1998; DeKlyen, Speltze, et al., 1998) found that father involvement favourably predicted children’s behaviour in a longitudinal study of clinic referred boys, over and above maternal characteristics. One of the challenges faced by research in this field is the variety of ways in which father involvement and fathering are defined and described. A widely used framework is that of Lamb and colleagues, who define three components to father involvement: engagement (or interaction), availability and responsibility (Lamb, Pleck, Charnov, & Levine, 1985). As more recent research on parenting has emphasised the importance of warmth and responsiveness in interaction, so revisions to this framework emphasising these factors, and the importance of interactions of a more intensive kind has come to the fore (Pleck, 2010). These detailed components of interaction can be assessed through diary accounts and questionnaire measures, but increasingly, observational methods, including detailed analyses of video-recorded interactions, have become the gold standard for assessing father-child as well as mother-child interactions.

The question arises as to how differences in paternal engagement or interaction with their children might impact on children’s development. There are a number of possible mechanisms that may account for the associations seen. Low levels of paternal engagement may reflect a less confident involvement of fathers in their children’s lives, and potentially more inconsistent or harsh parenting at other times. Children may thus have to work harder to engage the attention of their fathers, including through oppositional behaviour, resulting in negative patterns of interaction between child and parent, and consequently an increasing risk of oppositional behaviours. In contrast warm, engaged patterns of parenting allow the child to learn to manage emotional difficulties and positive strategies for dealing with difficulties ((DeKlyen, Biernbaum, et al., 1998; DeKlyen, Speltze, et al., 1998); Trautman-Villalba et al., 2006). Disengagement of fathers from their infants may also reflect wider dysfunction or conflict in the family or paternal psychopathology, such as depression or antisocial behavioural traits, any of which may lead to increased levels of behavioural disturbance in the child, through a mix of environmental and genetic mechanisms.

It is therefore important to attempt to better understand the relative contribution of fathers to their children’s early development. However, context is critical. Given that mothers are on average responsible for a greater proportion of child care than fathers, it is critical that research attempts to parse out the effects of maternal caregiving when examining how fathers involvement may affect children’s development. This is particularly the case, as not all studies find effects of paternal involvement when maternal care characteristics are controlled for.

Given the relative paucity of work on fathers and their interactions with their infants, and the importance of identifying children at risk of behavioural problems, study of these facets of early child development is worthy of sustained attention. The aim of the present study is to examine whether father–infant interaction in infancy (3 months postnatal) is associated with the early onset of child externalising behaviour (assessed at 1 year), and to identify whether specific aspects of these interactions are important in predicting behaviour. We hypothesised that children whose fathers’ were more engaged and sensitive/responsive in their interactions would have lower levels of behavioural problems, even controlling for important covariates, such as maternal sensitivity and paternal depressive and antisocial symptoms.

## Methods

### Participants and procedure

The present study was a longitudinal cohort study of fathers and their families. Participants were recruited from the postnatal maternity wards of the hospitals in Oxford and Milton Keynes. They were subsequently contacted and assessed at home at 3 months and 1 year postpartum.

Fathers participated in an initial screening assessment which included a depression questionnaire (the Edinburgh Postnatal Depression Scale – EPDS (Cox, Holden, & Sagovsky, 1987). The initial recruitment process has been described in more detail elsewhere (Ramchandani et al., 2011), but the aim was to recruit a sample weighted towards higher levels of depression. All fathers scoring 10 or above on the EPDS were invited to participate in the main study, along with a random 1 in 4 sample of low-scoring fathers. This approach yielded a sample of 192 families who were visited when the infant was 3 months old. Informed consent was obtained from all participants and then an assessment was undertaken which included filming of fathers interacting with their infant in two settings – a seat setting and a floor mat setting – for 3 min each.

In the seat setting, the infant was placed in an infant seat with the parent sitting facing them. For the second setting, the infant was placed on a floor mat on their back with the parent positioned face to face with their infant. For both interactions fathers were instructed to play with the infant in any way they chose without the use of toys or objects, for 3 min. Mothers were also filmed separately interacting with their infants in the seat setting for 3 min. The order of parental filming (mother/father) was counterbalanced. These video-recorded interactions were then coded by trained researchers who were blind to any of the participant characteristics.

Families were contacted again at 12 months postpartum. A total of 168/192 (87.5%) families participated in this second stage and completed questionnaire measures, including the Child Behavior Checklist (CBCL). Data on both interactions and child behaviour were available on 155 of these 168 families. The reasons for noncompletion were; incomplete questionnaires (3 families), and incomplete data on parent-child interactions (10 families).

There were no differences between those who did and did not complete the 1 year assessment in terms of infant gender, χ^2^ (1) = .504, *p* = .311, paternal academic qualifications, χ^2^ (5) = 2.48, *p* = .779, paternal age, *t*(189) = .218, *p* = .828, or paternal depressive status. There was a significant difference in socioeconomic status (SES) between the two groups, χ^2^ (3) = 22.2, *p* < .001. Families that completed the second part of the study were more likely to have professional occupations than noncompleters.

### Measures

#### Father–infant interactions

Father–infant interactions were assessed using the Global Rating Scales (GRS) (Murray, Fiori-Cowley, Hooper, and Cooper, 1996), a video-based assessment of the quality of parent-infant interaction. They were developed to assess differences between mothers with and without postnatal depression, and have since been successfully applied to a range of other settings (Gunning et al., 2004). They can be used from 2 to 6 months postpartum and have been found to predict child outcome at 18 months and 5 years (Murray, Fiori-Cowley, et al., 1996; Murray, Hipwell, Hooper, Stein, and Cooper, 1996). Parental behaviour is rated on four dimensions: sensitivity, intrusiveness, remoteness and behaviour relevant to depression (e.g. sad or tense). The videotaped interactions were scored by a trained researcher who had not been involved in the family visit. The reliability of the assessment is described in detail elsewhere (Sethna, Murray, Netsi, Psychogiou, and Ramchandani, under, review), but in brief, reliability levels were high for all dimensions, with intraclass correlations ranging from ICC = .74 to ICC = .88. The four scales are all scored from 0 to 5, with five generally representing more positive interactions; higher levels of sensitivity and lower levels of remoteness, intrusiveness and depressive behaviours. It is of note, however, that both low and high levels of intrusiveness are potentially negative predictors of child outcome, as low levels of intrusiveness can also represent a more withdrawn or disengaged style of interaction (Murray, Hipwell, et al., 1996). In these scales, remote interactions are those where a father is silent or not engaged with the infant. Intrusive interactions are those where the father interrupts the infants play through action or speech. Hence, a father who is being less intrusive may be not interrupting because he is being a more responsive father (a positive thing), or because he is less engaged overall in the interaction with his infant (a negative occurrence). Maternal and Paternal scores were found to be related on the Remote (*r* = 0.314; *p* < 0.01) and Depressive scales (*r* = 0.161; *p* = 0.04) but not on the Sensitivity (*r* = −0.009; *p* = 0.92) or Intrusiveness (*r* = 0.121; *p* = 0.13) scales.

#### Child Behavior CheckList (CBCL)

The CBCL is the most widely used questionnaire for the assessment of child behaviour problems. We used the version of the CBCL for ages 1½–5 years (Achenbach & Rescorla, 2000). Parents rate behaviours on a 3-point scale. This has previously been used with parents of one-year old children and found to be valid and to predict later behavioural problems (van Zeijl et al., 2006). We scored the same items to assess externalising problems under three subcategories: oppositional (17 items), aggressive (nine items) and overactive (five items). The three subcategories combine to give an overall externalising score.

Mothers and fathers completed the questionnaire independently. Internal consistencies (Cronbach’s alpha) for mothers and fathers were high for externalising (.89/.87) and oppositional (.85/.82) scales, and acceptable for overactive (.70/.66) and aggressive (.69/.62) scales. There was evidence of correlation between paternal and maternal CBCL ratings on all subscales, oppositional: *r* = .356, *p* < .001; aggressive: *r* = .190, *p* = .009; overactive: *r* = .323, *p* < .001, and on overall externalising behaviours, *r* = .341, *p* < .001. Maternal scores for behaviour were used for the analyses presented here, as we focus on early predictors relating particularly to fathers, and we wished to minimise reporter bias.

#### Other measures

##### Infant temperament

Infant temperament was measured on the inert-fretful infant scale of the GRS during mother-infant interactions. This scale runs from −2 (withdrawn) to +2 (fretful) and rates the infants’ attention to his environment, level of activity and affective state.

##### Maternal sensitivity

We selected maternal sensitivity as the key dimension to control for mother-infant interaction, as it is the domain of mother-infant interaction most consistently and strongly associated with adverse child outcome (Miner & Clarke-Stewart, 2008). This was measured by the sensitivity scale of the GRS during mother-infant interactions. This scale runs from 1 to 5 with a score of 5 representing the highest level of maternal responsiveness to her infant’s cues.

##### Parental depression

Parental depression was measured by use of the EPDS (Cox et al., 1987). This 10-item questionnaire has been extensively used in the perinatal period, and has high levels of sensitivity and specificity when used with men (Edmondson, Psychogiou, Vlachos, Netsi, & Ramchandani, 2010; Matthey, Barnett, Kavanagh, & Howie, 2001). Both parents also underwent a structured clinical interview to diagnose major depressive disorder.

##### Paternal antisocial behaviour

Fathers’ antisocial traits were measured with the Antisocial Personality Problems scale from the Adult Self-Report DSM-oriented scales. The scale consists of 20 items, with a response scale from 0 = Not true to 2 = Very true or often true. Internal consistency in the present study was 0.73.

### Statistical analyses

The analyses were undertaken in the following stages:

First, sample characteristics and demographics were examined, and are presented with means and standard deviations of continuous variables.

Second, bivariate correlations between the main father–infant interaction variables and the child behavioural variables were explored.

Third, hierarchical linear regression analyses were undertaken to control for the effect of potential confounding variables, where associations were found between elements of father–infant interaction and child behavioural outcomes. Covariates were selected that had previously been associated with behavioural problems. Paternal characteristics were included at Step 1 (SES, paternal age, depressive and antisocial symptoms). Step 2 included infant temperament and maternal sensitivity in mother-infant interactions and maternal depression. Father–infant interaction items were added at Step 3.

Fourth, any associations found were then re-examined in the sample split by infant gender, as previous research suggests that gender may be an important factor in outcomes for behaviour problems, with boys being more at risk than girls.

Fifth, and finally, to give an indication of the potential clinical relevance of these findings, binary variables were created for CBCL total externalising scores and scores on the remote and intrusive father-interaction scale, to allow us to assess the degree of risk in those with the most adverse early interactions. A cut-off score of 22 was used on the CBCL Total scale, with 10% of the sample obtaining scores above this point, indicating possible behavioural problems (Goodman & Scott, 1999). A cut-off score of 4 was used for both interaction variables, yielding 19.6% of the sample scoring below this on the remote scale, indicating remote interactions. On the intrusive scale, 26.6% of the sample scored above this point, indicating less intrusive, but more disengaged or withdrawn, interactions. A logistic regression model was undertaken to determine the odds of having behavioural problems given remote or nonintrusive interactions.

## Results

Demographic characteristics of the sample are presented in Table 1 and the means and standard deviations of the main predictor and outcome measures are shown in Table 2. The mean age of the fathers was 35 years, and they had higher than average levels of education. The sample contained approximately equal numbers of girls and boys.

**Table 1:** Characteristics of the study sample (*n* = 168)

	*N* (%) unless stated
Infant gender	
Male	78 (46%)
Female	90 (54%)
Paternal age (mean with *SD*)	35 (5.7)
Paternal academic qualifications^a^	
No qualifications	2 (1.2%)
GCSE	16 (9.5%)
A levels or equivalent	16 (9.5%)
Diploma or equivalent	28 (16.7%)
Degree	55 (32.7%)
Postgraduate	48 (28.6%)
SES	
Managerial/professional	95 (56.5%)
Intermediate occupations	45 (26.8%)
Routine/manual	27 (16.1%)
Unemployed	1 (0.6%)

a Data for three participants are missing.

**Table 2:** Descriptive statistics of study variables

	Mean (*SD*)
Maternal CBCL ratings	
Oppositional	8.29 (5.13)
Aggressive	1.57 (1.79)
Overactive	2.68 (1.95)
Externalising	12.55 (7.54)
Car seat interactions	
Sensitivity father	3.60 (0.55)
Intrusive father	3.86 (0.82)
Remote father	4.55 (0.84)
Depressive father	3.97 (0.61)
Good-poor infant	2.96 (1.05)
Inert-fretful infant	−0.14 (0.42)
Interaction	3.14 (0.96)
Floor mat interactions	
Sensitivity father	3.69 (0.55)
Intrusive father	3.70 (0.80)
Remote father	4.57 (0.83)
Depressive father	4.02 (0.54)
Good-poor infant	3.45 (1.08)
Inert-fretful infant	0.05 (0.35)
Interaction	3.46 (1.06)

CBCL, Child Behavior Checklist.

### Early father–infant interaction and later child behaviour

The correlations between father–infant interaction variables and child behavioural outcomes were examined separately for the interactions in the car seat setting and the floor mat setting. In the seat setting, none of the father interaction scales were associated with later child behavioural scores. However, in the floor mat setting, some evidence of negative correlation was found between the remote father scale and aggressive (*r* = −.218; *p* < .01) and overall externalising (*r* = −.143; *p* < .05) scores, indicating that more remote interactions were associated with higher CBCL scores. In addition, there were correlations between the intrusive father scale and oppositional (*r* = .157; *p* < .05) and overall externalising (*r* = .138; *p* < .05) scores, indicating that less intrusive (or more disengaged) interactions were associated with higher CBCL scores. The significance of these correlations should be considered cautiously in the light of the relatively large number of correlations undertaken.

After controlling for the effects of potential confounding variables (SES, paternal age and depressive and antisocial symptoms, infant temperament, maternal sensitivity and maternal depression), more remote father–infant interactions on the floor mat were still associated with a higher rate of aggressive behaviours (standardised β = −0.202; *p* = 0.02) and overall externalising behavioural problems (β = −0.175; *p* = 0.048) (see Table 3).

**Table 3:** Linear regression predicting child externalising problems at 1 year from fathers’ remote interactions

Factor	Standardised beta	*t*	*p*-value
Step 1			
Paternal age	−.214	−2.521	.013
Paternal employment	.001	.016	.987
Fathers depression	.102	1.163	.247
Fathers antisocial symptoms	−.031	−.347	.729
Step 2			
Maternal sensitivity	.017	.199	.842
Maternal depression	.060	.679	.498
Infant fretfulness	.087	.987	.326
Step 3			
Paternal remoteness	−.175	−2.001	.048

After controlling for the effects of confounding variables, the association between less intrusive (or more disengaged) paternal interactions and child oppositional behaviours attenuated slightly (β = 0.170; *p* = 0.05). Similarly, the association between less intrusive interactions and overall child externalising behaviours also attenuated (β = 0.141; *p* = 0.103) (see Table 4).

**Table 4:** Linear regression predicting child externalising problems at 1 year from fathers disengaged (less intrusive) interactions

Factor	Standardised beta	*t*	*p*-value
Step 1			
Paternal age	−.214	−2.521	.013
Paternal employment	.001	.016	.987
Fathers depression	.102	1.163	.247
Fathers antisocial symptoms	−.031	−.347	.729
Step 2			
Maternal sensitivity	.017	.199	.842
Maternal depression	.060	.679	.498
Infant fretfulness	.087	.987	.326
Step 3			
Paternal disengagement	.144	1.643	.103

### Gender differences

The association between remote father interactions and child aggressive behaviours was found in boys (β = −0.332; *p* = 0.01) but not in girls (B = −0.109; *p* = 0.38). Similarly, the association between remote father interactions and child externalising problems was found in boys (β = −0.408; *p* = 0.002) but not in girls (β = −0.001; *p* = 0.99). No gender differences were found for intrusive paternal interactions.

### Associations with higher levels of behavioural problems

When binary variables were created for high scores on father–infant interaction and externalising problems in children, to assess the potential clinical importance of any association, only weak evidence of an association between remote interactions and later child behaviour [Odds Ratio 2.46 (0.76, 7.94)] was found. This increased to 3.32 (0.92, 12.05) when covariates were controlled for. Stronger evidence was found for an association between less intrusive or more disengaged interactions and child behaviour [OR 3.15 (1.03, 9.61)], which again increased when covariates were included in the model (adj. OR 5.33 (1.39, 20.40) (see Table 5).

**Table 5:** Associations between father–infant interactions at 3 months, and high levels of behavioural problems in children at 1 year

Paternal factor	*N* (%) with behavioural problems in low risk group	*N* (%) with behavioural problems in high risk group	Odds ratio (95% Confidence Interval)	Adjusted OR (95% CI)^a^
Remote	9/124 (7.3)	5/31 (16.1)	2.46 (0.76, 7.94)	3.32 (0.92, 12.05)
Intrusive	7/114 (6.1)	7/41 (17.1)	3.15 (1.03, 9.61)	5.33 (1.39, 20.40)

a Adjusted for SES, fathers’ age and depressive symptoms, maternal sensitivity, maternal depression and infant temperament.

When boys and girls were analysed separately the associations appeared to be predominantly present in boys. However, the relatively small numbers in the groups when divided by gender rendered the estimates quite unstable and so it is difficult to draw firm conclusions.

## Discussion

The findings of this study suggest that key aspects of father–infant interaction, measured very early in children’s lives, are associated with an increased risk of behavioural problems in children at an early age. This is the first time that this apparent influence has been demonstrated for observed father–infant interaction and such early onset behaviour problems. The association is independent of other key risk factors, including maternal sensitivity, difficult infant temperament and paternal characteristics, including depression and antisocial symptoms. The aspects of interaction which are the key predictors of child behavioural problems are more remote and less intrusive interactions. These share the similar characteristic that they both reflect more disengaged interaction on the father’s part, although they reflect somewhat different components of interaction. Thus, remoteness includes being lost in one’s own thoughts, so being more psychologically detached, whereas the intrusive dimension comprises physical and verbal behaviours to interrupt the infant, and so assesses a different form of engagement. The two scales are negatively correlated with each other (*r* = −.247, *p* = .018), although the relatively modest level of this correlation suggest that the scales are assessing different aspects of interaction. Overall, the pattern of association was stronger for boys than for girls, raising the intriguing possibility that boys may be more susceptible to the influence of their father from a very early age. Before considering how these findings fit with existing literature and their possible clinical implications, several limitations and strengths of the research must be considered.

The study has a number of important strengths. It uses a longitudinal design to assess associations over the first year of a child’s life. It is increasingly recognised that the origins of behavioural problems in many children lie in their very early development. In addition, an observational measure of father-infant interaction was used, which was coded in a blind manner by trained researchers. We were also able to control for the important potential effects of mother-infant interaction. Finally, a relatively low level of attrition occurred over the 9 months of the study (approximately 13%).

There are, however, some limitations to consider. The sample of fathers was somewhat older and had a higher level of education than that of the population from which it is drawn. This occurred despite recruiting from widely used maternity units in the local health service. Hence, these findings should be generalised to the whole UK population cautiously. Second, the assessment of child behaviour was undertaken at a young age. Although the CBCL has previously been used with children as young as 1 year (van Zeijl et al., 2006), it is not yet a widely used and validated measure of behaviour at this age. In addition, it was assessed by parental report, although we used maternal report to minimise single-reporter bias. Parental report is the commonest form of behavioural assessment at such a young age and predicts later outcome well (Fergusson, Boden, & Horwood, 2009). Nevertheless, an observational measure of behaviour would be a useful addition for future studies. Third, for the estimation of odds ratios, the numbers of participants in the high risk groups for paternal interactions and child behaviour were relatively small, leading to wide confidence intervals around the estimates of effects. Finally, although the study employed a longitudinal design, the associations should not be read as demonstrating a causal relationship between father–infant interaction and child behavioural problems.

A number of previous studies have found similar associations between aspects of fathering and later child development, although these have tended to be in older children. Overall father involvement has been found to be associated with a range of positive outcomes for children (Sarkadi et al., 2008). More specifically, paternal sensitivity and warmth in interactions has been demonstrated to predict a lower level of behavioural disorder much later in childhood (Amato & Rivera, 1999; Trautman-Villalba et al., 2006). However, the current study is the first to assess observed father–infant interaction and behavioural problems within the first year of life. There are a number of possible explanations for the apparent importance of remoteness or disengagement of fathers from these early interactions. This lack of paternal engagement could reflect a wider dysfunction in family relationships, with fathers who are in a more troubled relationship with their partner finding it more challenging to engage with their infant. This may be because they have had more limited experience in playing with or otherwise interacting with their infant, or perhaps because it evokes more negative emotion in themselves, and so is a more aversive experience for them. There is some evidence that mothers can act in a gatekeeper role in some families, and that difficulties in couple relationships can lead to fathers having reduced opportunity to engage with their children (Schoppe-Sullivan, Brown, Cannon, Mangelsdorf, & Sokolowski, 2008). A second possible explanation is that the lack of engagement in the observed interaction reflects a broader lack of supervision and potentially care, for the infant, resulting in an increase in behavioural disturbance. Third, it is possible that the behavioural disturbance in the infant represents an attempt to elicit a parental reaction in response to an earlier lack of parental engagement. It is likely that all of these processes play some part, and that an interactive process between a father and the infant takes place over time.

The finding that these associations appear more pronounced in boys is somewhat surprising given the very young age of the children. There is limited work examining gender differences in relation to fathers influence. Some previous research found that paternal depression was associated with increased behavioural problems in young boys but not girls (Ramchandani et al., 2005). However, father engagement later in childhood predicts better educational outcomes for girls in particular (Flouri & Buchanan, 2004). There is little consistent evidence on which to draw, and possible explanations are somewhat elusive. Some work has suggested that fathers may interact differently with their sons compared with daughters (Lewis & Lamb, 2003), but it is not clear that such a difference would necessarily be in operation with children as young as 3 months old. There are studies pointing to an increased sensitivity in boys to the effects of maternal parenting (Martin, 1981; Rothbaum & Weiss, 1984; Shaw & Gross, 2008), which may point to some common pathways or an overall greater vulnerability in boys. Further work is needed, both to see if the association with paternal engagement is a consistent association and, if it is, to explore what the possible explanations might be.

One further issue to consider is the method employed to assess father–infant interaction. A conventional interaction task was used, adapted from studies of mother-infant interaction (Murray, Fiori-Cowley, et al., 1996). This employed the use of a seat for the infant to sit in, with the father sitting directly in front. After initial piloting, we found that fathers found this quite a challenging situation, and it appeared to restrict the use of strategies that fathers are found to traditionally employ in their interactions with young children, including more physical play and active engagement. We therefore introduced a second interaction setting (the floor mat setting) which allowed more freedom to move for both the father themselves, and for them to move their infant. It is notable that the associations found with later behavioural development were found in the floor mat setting but not in the car seat setting. This is an important consideration for research on fathers and fathering, which has often borrowed measures that were originally developed for mothers. Novel, theoretically driven assessments of father–infant behaviour are needed if potentially important aspects of father-child interaction and paternal influence are not to be missed.

The findings extend the current field of research by demonstrating an association between father–infant interaction and child behavioural problems early in life. This focus on the earliest stages of development is important, as the early postnatal period, along with foetal development during pregnancy, represents a period of crucial development, when developmental plasticity is potentially at its greatest (Bateson et al., 2004; Glover, 2011) and the infant is most susceptible to environmental influences, such as the quality of parental care and interaction. Some possible clinical implications are raised by these findings. Behavioural problems are the commonest form of psychopathology affecting children, and they consistently predict a wide range of adverse outcomes for children as they progress to adolescence and early adult life. Whereas interventions with beneficial effects in the treatment of behavioural problems do exist for older children (most notably parenting programmes), many behavioural problems prove relatively intractable to treatment once established. Early parent-child interactions may represent an important potential point of intervention. Preventive interventions targeted at causal pathways of risk are strong candidates for effective, and cost-effective, amelioration of some of these difficulties in families at risk. The contribution of the present study is to highlight that the interactions of fathers, as well as those of mothers, may be critical factors to address, from a very early age in the child’s life.

## Key Points

- The quality of parents’ early interactions with their children is an important predictor of the development of behavioural problems in children. However, relatively few studies have addressed the role of fathers, despite this appearing to be particularly pertinent to child behavioural development.
- This study found that disengaged and remote interactions between fathers and their infants at age 3 months independently predicted externalising behavioural problems at the age of 1 year.
- These interactions may be critical factors to address, from a very early age in the child’s life, and offer an opportunity for potential preventive intervention.
